# Clinical and population‐based study design considerations to accelerate the investigation of new antiretrovirals during pregnancy

**DOI:** 10.1002/jia2.25917

**Published:** 2022-07-19

**Authors:** Sean S. Brummel, Jeff Stringer, Ed Mills, Camlin Tierney, Ellen C. Caniglia, Angela Colbers, Benjamin H. Chi, Brookie M. Best, Myriam El Gaaloul, Sharon Hillier, Gonzague Jourdain, Saye H. Khoo, Lynne M. Mofenson, Landon Myer, Sharon Nachman, Lynda Stranix‐Chibanda, Polly Clayden, Memory Sachikonye, Shahin Lockman

**Affiliations:** ^1^ Department of Biostatistics Center for Biostatistics in AIDS Research Boston Massachusetts USA; ^2^ Harvard T.H. Chan School of Public Heath Boston Massachusetts USA; ^3^ School of Medicine University of North Carolina Chapel Hill North Carolina USA; ^4^ MTEK Sciences Vancouver British Columbia Canada; ^5^ MTEK Sciences Kigali Rwanda; ^6^ Perelman School of Medicine University of Pennsylvania Philadelphia Pennsylvania USA; ^7^ Department of Pharmacy Radboud Institute for Health Sciences Radboud University Medical Center Nijmegen The Netherlands; ^8^ Department of Obstetrics and Gynecology University of North Carolina at Chapel Hill Chapel Hill North Carolina USA; ^9^ Skaggs School of Pharmacy and Pharmaceutical Sciences San Diego California USA; ^10^ Pediatrics Department – Rady Children's Hospital San Diego University of California San Diego La Jolla California USA; ^11^ Product Development Medicines for Malaria Venture Geneva Switzerland; ^12^ Department of Obstetrics Gynecology and Reproductive Sciences University of Pittsburgh and the Magee‐Womens Research Institute Pittsburgh Pennsylvania USA; ^13^ MIVEGEC University Montpellier Montpellier France; ^14^ Department of Pharmacology University of Liverpool Liverpool UK; ^15^ Research Department Elizabeth Glaser Pediatric AIDS Foundation Washington DC USA; ^16^ Division of Epidemiology & Biostatistics School of Public Health & Family Medicine University of Cape Town Cape Town South Africa; ^17^ Department of Pediatrics The State University of New York (SUNY) Stony Brook New York USA; ^18^ Child and Adolescent Health Unit Faculty of Medicine and Health Sciences, University of Zimbabwe Harare Zimbabwe; ^19^ HIV i‐Base London UK; ^20^ Brigham and Women's Hospital Boston Massachusetts USA

**Keywords:** ARV, clinical trials, intervention, paediatrics, treatment, viral suppression

## Abstract

**Introduction:**

Pregnant women are routinely excluded from clinical trials, leading to the absence or delay in even the most basic pharmacokinetic (PK) information needed for dosing in pregnancy. When available, pregnancy PK studies use a small sample size, resulting in limited safety information. We discuss key study design elements that may enhance the timely availability of pregnancy data, including the role and timing of randomized controlled trials (RCTs) to evaluate pregnancy safety; efficacy and safety outcome measures; stand‐alone protocols, platform trials, single arm studies, sample size and the effect that follow‐up time during gestation has on analysis interpretations; and observational studies.

**Discussion:**

Pregnancy PK should be studied during drug development, after dosing in non‐pregnant persons is established (unless non‐clinical or other data raise pregnancy concerns). RCTs should evaluate the safety during pregnancy of priority new HIV agents that are likely to be used by large numbers of females of childbearing age. Key endpoints for pregnancy safety studies include birth outcomes (prematurity, small for gestational age and stillbirth) and neonatal death, with traditional adverse events and infant growth also measured (congenital anomalies are best studied through surveillance). We recommend that viral efficacy be studied as a secondary endpoint of pregnancy RCTs, once PK studies confirm adequate drug exposure in pregnancy. RCTs typically use a stand‐alone protocol for new agents. In contrast, master protocols using a platform design can add agents over time, possibly speeding safety data ascertainment. To speed accrual, stand‐alone pregnancy trial protocols can include pre‐specified starting rules based upon adequate PK levels in pregnancy; and seamless master protocols or platform trials can include a pregnancy PK and safety component. When RCTs are unethical or cost‐prohibitive, observational studies should be conducted, preferably using target trial emulation to avoid bias.

**Conclusions:**

Pregnancy PK needs to be obtained earlier in drug evaluation. Timely RCTs are needed to understand safety in pregnancy for high‐priority new HIV agents. RCTs that enrol pregnant women should focus on outcomes unique to pregnancy, and observational studies should focus on questions that RCTs are not equipped to answer.

## 1 INTRODUCTION

Pregnancy and lactation data are lacking for more than 90% of Food and Drug Administration (FDA)‐approved drugs [1], and pregnant/breastfeeding women are routinely excluded from pre‐ and post‐licensure clinical trials [2] (although pregnancy and breastfeeding have overlapping issues, the primary focus of this commentary is on pregnancy). Pregnancy data for antiretrovirals (ARVs) are usually limited to pregnancy pharmacokinetics (PK) with minimal safety information, collected in small, delayed “opportunistic” PK studies that enrol pregnant women who are taking approved HIV drugs in clinical care settings [2, 3]. The median time between new drug approval and the first published PK data in pregnancy is 6 years [4]. It is rare to have pre‐licensure trials intentionally enrol pregnant women to study the safety and efficacy (for viral suppression if used in treatment, or for reduction of HIV risk if used for prevention) of ARVs during pregnancy [5, 6, 7, 8]. Absent or delayed pregnancy data can cause significant harm by limiting our evidence‐based treatment options, resulting in the prolonged use of regimens during pregnancy that may be less potent, less convenient and less tolerated, or by using newer regimens with unknown toxicity or pregnancy PK [9].

To address these issues, the IMPAACT Network and World Health Organization (WHO) convened a workshop, as described in Penazzato et al. [10], which included in‐depth discussions around trial design to support the accelerated investigation of new HIV agents to treat and prevent HIV in pregnant women. Building on those discussions, we suggest approaches to study design and implementation to more efficiently conduct ethical and timely research of new HIV treatment and prevention agents during pregnancy. We identify key outcome measures of pregnancy trials and their ascertainment and definition. We then discuss considerations in designing randomized controlled trials (RCTs) in pregnancy and alternative designs that may improve efficiency, such as studies that integrate different trial phases into one seamless platform trial, and touch upon other design considerations, such as sample size. We end by outlining the role of observational studies of HIV drugs in pregnancy. Our overarching goal is to support ethical research that will provide comprehensive and timely pregnancy data for new ARVs used for HIV treatment or prevention, so that females and their healthcare providers have high‐quality data to inform decisions regarding their care in pregnancy.

## 2 DISCUSSION

### 2.1 Clinical safety outcome measures

Safety outcomes for drugs used in pregnancy are of primary importance. Safety evaluations should focus on safety outcomes that are uniquely important to mothers and infants as described below—for example birth outcomes, neonatal mortality, infant growth and specific maternal adverse events that may differ between the pregnant and non‐pregnant state, such as gestational diabetes, hepatotoxicity or maternal neuropsychiatric concerns (Table 1). While general safety analyses of adverse events are often summarized in RCTs (e.g. occurrence of any Grade 3 or higher severity adverse event) and should be collected, we recommend that analyses of general adverse events be studied as secondary outcome measures.

**Table 1:** Key safety outcome measures for safety pregnancy studies

Outcome	Considerations
Preterm birth (PTB)	PTB is defined as delivery of a live‐born foetus *prior to 37 weeks of gestation*, complicates 15 million pregnancies each year and is associated with neonatal death and disability [11, 12, 13, 14]. Classification of PTB requires accurate estimation of gestational age. It is common in low‐ and middle‐income countries (LMICs) to estimate gestational age using methods that are subject to error and bias (e.g. last menstrual period, symphysis‐fundal height or newborn examination) [15, 16]. To accurately ascertain PTB, research *protocols should include foetal ultrasound for dating*, ideally in the first trimester.
Small for gestational age (SGA)	SGA is defined as sex‐specific *weight‐for‐age at birth <10th centile* [17, 18] and affects more than 20 million births per year in LMICs. SGA may result from intrauterine growth restriction or represent non‐pathologic variation. For this reason, the *third centile of weight‐for‐age at birth may represent a preferable definition* for research purposes, as it is more specific for pathology and associated with higher morbidity/mortality [19].
Foetal loss	Foetal loss before 20 weeks’ gestation is defined as *spontaneous abortion*; this endpoint is most relevant to surveillance. *Stillbirth*, defined as the *in utero* foetal death of a foetus after 20 weeks’ gestation by CDC [20] and 28 weeks by WHO [21], affects 2.6 million pregnancies annually [22]. *Ultrasound dating is helpful to ensure correct classification* of foetal loss.
Neonatal mortality	The causes of observed differences in neonatal mortality by antiretroviral regimen in some studies are unclear [23, 24]. Research studies should *include neonatal mortality as an outcome, with the cause of death* (and for high‐priority agents, power studies to detect at least moderate differences in neonatal mortality).
Congenital anomalies	While congenital anomalies related to medications in pregnancy are a concern, *true teratogens are rare* [25]. Approximately 3–6% of babies in the United States are born with a serious congenital anomaly [26]. Ascertainment and understating of the rate of congenital anomalies varies widely by country with a dearth of information in Africa [27]. Surveillance studies are necessary for understanding the associations of new agents with congenital anomalies [28]. Where congenital anomalies are reported, we recommend: (1) *using a pre‐specified definition of anomaly* [29]; (2) *conducting systematic and standardized anomaly assessment* [30] and (3) including *expert adjudication* of reported anomalies blinded to treatment.
Other outcomes	Low birthweight (LBW, <2500 g) is an easily ascertained birth outcome commonly used in research [31] but can be *problematic* because it conflates the pathologically distinct processes of preterm birth and small for gestational age. For that reason, *PTB and SGA* should ideally be considered separately in lieu of LBW. Several other important maternal and infant outcomes should be studied in the postpartum period (including during breastfeeding). While a thorough discussion of these measures is beyond the scope of this manuscript, we would highlight *infant growth (ideally through 1 year of age)* as a readily measured additional outcome that should be included as a secondary endpoint in RCTs. Summarizing growth may help to understand the possible longer term effects of study drug on prematurity [32].

The table details many pregnancy safety outcome measures to study. We suggest the use of a composite endpoint—defined as the occurrence of any adverse birth outcome of interest—that is of public health relevance, with the aim to optimize overall birth and neonatal outcomes. We believe that additional thought should be dedicated to delineating which specific adverse birth outcomes should be included in a composite endpoint, and its relevance to the study population. However, we suggest that in general, the composite birth outcome should include prematurity (<37 weeks), SGA (third percentiles) and foetal loss. All three are clinically important; foetal loss may cause live‐birth bias of prematurity and SGA estimates [33, 34]. Each outcome type should also be analysed separately to understand its relative contribution and direction of association with study treatment. With additional analyses that include neonatal death and congenital anomalies, the full set of analyses will facilitate selecting the agent with the greatest chance of a healthy baby.

### 2.2 Efficacy

For ARVs with high virologic efficacy demonstrated in antiretroviral treatment (ART) trials in non‐pregnant women and for which PK exposure in pregnancy is adequate, one would expect similar efficacy in pregnancy; this is expected to also be the case for most ARVs used for prevention. There is thus limited rationale to repeat treatment or prevention efficacy trials solely on the basis of pregnancy. When ART is started early enough in (or before) pregnancy and adherence is high, vertical transmission is rare [7, 24], making it infeasible to design trials with vertical transmission as a primary endpoint. We thus recommend that viral suppression and vertical transmission (or HIV incidence, for pre‐exposure prophylaxis) in pregnancy be at least descriptively compared between arms in secondary analyses of trials of ARVs in pregnancy, unless a specific rationale warrants studying efficacy as a primary outcome measure. For example, an efficacy study might be considered for pregnant women presenting late in gestation to evaluate the rapidity of viral suppression [35].

### 2.3 Study design recommendations

#### 2.3.1 Randomized controlled trial

RCTs with blinded active or placebo control allow for direct comparison and reduce selection, allocation, investigator/participant biases and unobserved confounding. RCTs are also important when evaluating the pregnancy safety of new agents, especially for birth outcomes. “Background” rates of birth outcomes vary substantially by many factors, which may differ by population, location and time, including maternal age, parity, obstetric history, socio‐economic, nutritional status, quality of local care and outcome ascertainment method. In addition, predictors of birth outcomes are not well understood, resulting in the potential for unobserved confounding. These factors taken together make it difficult to fully measure and control for all confounders when comparing birth outcomes with a new agent to a non‐randomized control group. Hence, single‐arm pregnancy trials of new agents (that compare safety outcomes with the study agent to “background” rates of these outcomes) are essentially observational in nature. As such, while we acknowledge that large pregnancy safety RCTs are not feasible or warranted for all new agents, we recommend the use of RCTs to evaluate the safety of new high‐priority HIV agents compared to either standard of care or to other ARV regimens with an optimal safety record [36]. Ideally, such dedicated pregnancy safety studies would start during phase 3 pre‐registrational trials or early post‐approval (assuming pre‐clinical studies and safety data in non‐pregnant people do not raise concerns, and after adequate pregnancy PK has been confirmed). It is also important to conduct these RCTs in a variety of settings and populations for whom the results will be most relevant.

#### 2.3.2 Alternative RCT designs to shorten timelines and enhance efficiency

Randomized designs that can shorten the time to obtain pregnancy safety data should be considered. The standard option is a stand‐alone protocol, where an RCT aims to answer a select number of questions about the effect of an ARV regimen in a specific population. These designs are well understood and reflect interventions available at a specific time [37]. Importantly, stand‐alone protocols do not typically accommodate the study of new regimens that may arise during the trial. This perhaps explains some of the delays in the reporting of high‐quality randomized pregnancy data. If a stand‐alone protocol is used for an RCT in pregnancy, we recommend that the protocol includes pre‐specified starting rules, including cutoffs for adequate pregnancy PK levels (potentially obtained from sources other than the study itself), so the pregnancy safety study can start as soon as possible.

In 2018, the US FDA provided guidance on master protocols. Generally, these are protocols that include multiple studies that require coordination to evaluate multiple drugs or multiple study populations [33]. Master protocols encompass adaptive clinical trials [34, 38, 39] and fixed sample designs, the incorporation of real‐world evidence, and the necessity for long‐term planning of clinical research portfolios. Three approaches to master protocols have received support and guidance from the FDA [40]. These include basket trials, umbrella trials and platform trials. Platform trials may be the most applicable to studying safety in pregnancy as they allow for continual evaluation of multiple interventions for a single disease or condition. Platform trials are perpetual multi‐arm trials that may add or drop arms based on accruing internal and external evidence [40, 41]. New arms can be added to platform trials as they become available over time and a common comparison group can be updated to reflect changes in standard of care.

Researchers should consider a safety platform trial using a master protocol for studies in pregnancy. Platform trials can introduce additional complexities, but seamless designs, which include a PK assessment and a subsequent safety investigation, could expedite gathering pregnancy safety data, particularly if the design of the second phase is a simple randomization. Ideally, a platform trial would include two phases for each new agent. The first phase would focus on obtaining PK (with safety data in a small number of people) in pregnancy with thresholds for when to start the next phase of the study. The second phase would be a phase 3 RCT safety study that compares the new therapy to a predefined comparison group. We anticipate that a platform trial with a seamless transition from a PK study to a phase 3 RCT would provide timely reporting of safety data to help inform guidelines. This platform trial could expedite reporting since the study infrastructure, including approval by regulatory bodies, protocol, data collection instruments and other necessary documentation, could be in place well before the completion of the pre‐licensure phase 3 study in non‐pregnant people.

#### 2.3.3 Detecting safety signals: considerations related to sample size, gestational age at enrolment

Sample size dictates the precision of the conclusions drawn from a study and the required resources. The typical sample size of pregnancy PK studies is between 12 and 25 females, depending on the variance in PK [42]. This sample size is not sufficient to give a full understanding of safety in pregnancy. As an example, consider a study with an active comparator that has an expected composite pregnancy outcome percentage of 30%. Also, assume an “acceptable” increase in the outcome of 12.5% with the study agent. Using a 95% confidence interval for the difference in probabilities with sufficient precision to either include the value of no difference or the acceptable increase, the required sample size would be 447 participants per arm—much larger than in PK studies. This example shows how underpowered PK studies are to understand safety in pregnancy. In addition, a larger sample size is required to rule out smaller differences, which are of scientific interest when studying rare outcomes. When the sample size is prohibitively large for an RCT, observational or surveillance studies are needed.

For studies in pregnancy, exposure to ARVs in different trimesters also affects conclusions drawn from the study. Rates of the birth outcomes described above vary by the amount of observed follow‐up at specific gestational ages. Studies that enrol participants with insufficient exposure earlier in gestation may not be sensitive for detection of the full safety effects of the study regimen. For example, organogenesis during the early first trimester is the period of greatest sensitivity to teratogens. Clinical trials have often limited randomization to pregnant women later in gestation (e.g. ≥ 14 weeks gestation), which limits the external validity of clinical trials [43]. Similarly, some clinical trials include an upper limit on the gestational age at enrolment, which also affects the interpretability of the observed rates of birth outcomes. We recommend careful consideration of the targeted *gestational age at study entry* when defining this key study criterion in RCTs. External validity can be expanded using observational or surveillance studies, since a representative sample of ART exposure in pregnancy can be ascertained.

#### 2.3.4 Role of observational studies and surveillance

Ideally, questions about the safety (and effectiveness, if in question in rare instances) of HIV‐related drugs in pregnancy would be answered with a well‐powered RCT. However, such trials are not always feasible or ethical, will almost never include early first trimester exposure, may have been conducted with a sample size insufficient to detect differences in rare events and may warrant confirmation in “real‐world” settings given external validity concerns that can arise with RCTs. For example, a trial evaluating the safety of a drug started before conception would require individuals to be enrolled and randomized before conception, become pregnant after randomization and followed throughout pregnancy.

When RCTs are not possible or rare outcomes are of interest, surveillance or observational study data should be used. Pregnancy surveillance approaches are discussed by Renaud et al. [28]. An additional promising approach is the use of observational data to emulate a hypothetical RCT, or target trial, that researchers would like to conduct to answer the research question of interest [44]. Target trial emulation is a two‐step process that first outlines the hypothetical RCT we would conduct to answer the research question (i.e. the eligibility criteria, treatment strategies, treatment assignment, outcomes, start and end of follow‐up, causal contrasts of interest and analysis plan). This process helps researchers avoid common biases often found in observational study design and analysis, while being transparent about which components of the target trial may not have been successfully emulated [45]. Target trial emulation requires large observational datasets with rich data. Ascertainment of these data will allow target trial emulation to complement or extend randomized trials by allowing researchers to study more generalizable populations.

## 3 CONCLUSIONS

Pregnancy PK data are often not available (or are markedly delayed) for new agents. Furthermore, randomized clinical trials are the gold‐standard approach to assessing safety risk of new agents but are rarely conducted in pregnant populations. We believe this has done a great disservice to the many patients, providers and regulatory bodies who are faced with weighing the relative risk and benefit of different ARV regimens for which minimal pregnancy data exist. In the current landscape of HIV treatment, the benefit of ART to the mother's health and ability of ART to reduce vertical transmission is well established. Pregnancy PK should be studied for all new HIV agents. When a pre‐licensure trial has demonstrated virologic efficacy in non‐pregnant people and PK in pregnancy is adequate, the efficacy of ARVs can be extrapolated to pregnant women. For new high‐priority HIV agents, pregnancy safety (in particular birth outcomes) should be studied as the primary outcome in RCTs, and done so with a sufficient sample size. Vertical transmission and viral suppression should be studied secondarily. Seamless designs (integrating pregnancy PK and larger safety trials) and pregnancy platform trials are an alternative to the current use of stand‐alone clinical trials, which may speed ascertainment of safety data in pregnancy. These pregnancy safety studies can be ethically started at the timely conclusion of non‐clinical developmental and reproductive toxicology and pregnancy, PK studies (in non‐pregnant and pregnant people) and once sufficient safety data in non‐pregnant people have been generated in a registrational study. In addition, surveillance systems (and observational studies, preferably using trial emulation) will be needed to answer questions about first trimester exposure, rare and longer‐term outcomes, and key subgroups.

It is only when pregnancy data are collected and reported with sufficient precision and in a timely manner that risk and benefit, a cornerstone to evaluate treatment options, can be adequately judged and used to optimize the care of pregnant women.

## COMPETING INTERESTS

AC has received honoraria from Merk Sharp & Dohme Corp 2021, and fee is paid to the institution. SH has received consulting fees from Merck, ViiV and Gilead, and her institution has received funding from Merck and Gilead.

## AUTHORS’ CONTRIBUTIONS

The concept for this commentary was conceived by all authors (SSB, JS, EM, CT, ECC, AC, BHC, BMB, MEG, SH, GJ, SHK, LMM, LM, SN, LSC, PC, MS and SL). SSB and SL created the outline for this commentary. First drafts of various sections of the manuscript were written by SSB, JS, EM, CT, EC, AC and SL. All authors (SSB, JS, EM, CT, EC, AC, BHC, BMB, MEG, SH, GJ, SHK, LMM, LM, SN, LSC, PC, MS and SL) provided comments and edits on the initial draft, and approved the final manuscript.

## FUNDING

SL was supported by K24 AI131928. SSB, CT and SN: Overall support for the International Maternal Pediatric Adolescent AIDS Clinical Trials Network (IMPAACT) was provided by the National Institute of Allergy and Infectious Diseases (NIAID) with co‐funding from the Eunice Kennedy Shriver National Institute of Child Health and Human Development (NICHD) and the National Institute of Mental Health (NIMH), all components of the National Institutes of Health (NIH), under Award Numbers UM1AI068632‐15 (IMPAACT LOC), UM1AI068616‐15 (IMPAACT SDMC) and UM1AI106716‐15 (IMPAACT LC), and by NICHD contract number HHSN275201800001I.

## DISCLAIMER

The content is solely the responsibility of the authors and does not necessarily represent the official views of the NIH.
